# The physicochemical properties of lipopolysaccharide chemotypes regulate activation of the contact pathway of blood coagulation

**DOI:** 10.1016/j.jbc.2024.108110

**Published:** 2024-12-18

**Authors:** André L. Lira, Berk Taskin, Cristina Puy, Ravi S. Keshari, Robert Silasi, Jiaqing Pang, Joseph E. Aslan, Joseph J. Shatzel, Christina U. Lorentz, Erik I. Tucker, Alvin H. Schmaier, David Gailani, Florea Lupu, Owen J.T. McCarty

**Affiliations:** 1 Department of Biomedical Engineering, Oregon Health & Science University, Portland, Oregon, USA; 2 Cardiovascular Biology Research Program, Oklahoma Medical Research Foundation, Oklahoma City, Oklahoma, USA; 3 Knight Cardiovascular Institute, Oregon Health & Science University, Portland, Oregon, USA; 4 Division of Hematology and Medical Oncology, School of Medicine, Oregon Health & Science University, Portland, Oregon, USA; 5 Aronora, Inc, Portland, Oregon, USA; 6 Division of Hematology and Oncology, Department of Medicine, Case Western Reserve University and University Hospitals Cleveland Medical Center, Cleveland, Ohio, USA; 7 Department of Pathology, Microbiology, and Immunology, Vanderbilt University Medical Center, Nashville, Tennessee, USA

**Keywords:** lipopolysaccharides, factor XII, contact pathway, coagulation, sepsis

## Abstract

Lipopolysaccharide (LPS) is the primary pathogenic factor in Gram-negative sepsis. While the presence of LPS in the bloodstream during infection is associated with disseminated intravascular coagulation, the mechanistic link between LPS and blood coagulation activation remains ill-defined. The contact pathway of coagulation—a series of biochemical reactions that initiates blood clotting when plasma factors XII (FXII) and XI (FXI), prekallikrein (PK), and high molecular weight kininogen interact with anionic surfaces—has been shown to be activated in Gram-negative septic patients. In this study, using an *in vivo* baboon model of Gram-negative *Escherichia coli* sepsis, we observed activation of the contact pathway including FXII, FXI, and PK. We examined whether *E.coli* LPS molecules could bind and activate contact pathway members by quantifying the interaction and activation of either FXII, FXI, or PK with each of the three chemotypes of LPS: O111:B4, O26:B6, or Rd2. The LPS chemotypes exhibited distinct physicochemical properties as aggregates and formed complexes with FXII, FXI, and PK. The LPS chemotype O26:B6 uniquely promoted the autoactivation of FXII to FXIIa and, in complex with FXIIa, promoted the cleavage of FXI and PK to generate FXIa and plasma kallikrein, respectively. Furthermore, in complex with the active forms of FXI or PK, LPS chemotypes were able to regulate the catalytic activity of FXIa and plasma kallikrein, respectively, despite the inability to promote the autoactivation of either zymogen. These data suggest that the procoagulant phenotype of *E.coli* is influenced by bacterial strain and the physicochemical properties of the LPS chemotypes.

Sepsis is defined as a life-threatening organ dysfunction caused by a dysregulated host response to infection (1, 2). The cardiovascular system is significantly impacted by sepsis, resulting in hypotension, systemic inflammation, cardiomyopathy, multi-organ failure, and coagulation issues like disseminated intravascular coagulation (DIC) (2, 3, 4, 5). Sepsis presents the host’s response to an infection or to circulating bacterial products, not just the infection itself. Gram-negative bacteria produce sepsis and septic shock in part *via* the release of cell envelope components including lipopolysaccharide (LPS).

LPS, which makes up about 75% of the outer membrane of Gram-negative bacteria, is a chemically stable glycolipid composed of three structurally and functionally distinct regions: a conserved phosphoglycolipid (lipid A moiety), a core oligosaccharide (core OS region; divided into inner and outer cores), and an O-polysaccharide chain (O-Antigen; or OAg) (6, 7, 8). The conserved lipid A and inner core region are highly anionic due to multiple negative charges from phosphates and carboxylates groups (9). The solubility of LPS is largely dependent on the hydrophilic properties of the OAg region, which forms a hydrophilic layer on the bacterial surface. As an amphipathic molecule, LPS typically forms negatively charged self-aggregates when released from the cell surface into aqueous solutions (10, 11, 12). The differences in physicochemical and antigenic properties of LPS between bacterial strains influence their overall pathogenicity, including capacity to evade the host immune system. These differences are in part related to the variance in the length, structure, and presence of the O-antigen region (13, 14). Bacteria that express LPS capped with a long OAg are classified as smooth strains (or S-LPS) while those lacking the OAg are considered rough strains (or R-LPS). Bacteria that express LPS chemotypes which contain only one repeating OAg subunit are classified as semi-rough strains (or SR-LPS) (6). How or even whether the physicochemical distinction between LPS chemotypes manifest as pathological differences in maladies ranging from organ failure to thrombosis remains unknown.

Bacterial components, including LPS, are known to trigger the activation of the coagulation cascade, acting in part as an extension of innate immunity to restrict dissemination of bacteria within the bloodstream (15). The coagulation cascade involves a complex series of biochemical reactions whereby inactive zymogens are converted to active serine proteases. The coagulation cascade can be triggered by extracellular matrix proteins exposed by vessel injury as part of the extrinsic pathway of coagulation (16) or by negatively charged foreign surfaces as part of the contact pathway of coagulation (17, 18). The contact pathway traditionally consists of three serine zymogens: coagulation factors XII (FXII) and XI (FXI), plasma prekallikrein (PK), and the nonenzymatic cofactor high molecular weight kininogen (HK). As blood comes into contact with anionic surfaces such as polymers (19, 20, 21) and pathogenic microorganisms (22) or nonbiological materials including silicates (23, 24), biomedical artificial surfaces (25), and metals (26, 27), the zymogen FXII is activated to form the active protease factor XIIa (FXIIa). FXIIa converts PK to the protease plasma kallikrein (PKa), which, in turn, converts FXII to FXIIa in a positive feedback loop. FXIIa cleaves FXI to form FXIa, which then activates factor IX (FIX) to FIXa to promote clot formation (28, 29, 30). Circulating in complex with FXI and PK, HK acts as a cofactor for PK and FXI activation by FXIIa (31, 32).

We showed that pharmacological inhibition of FXII activation and FXIIa activity prevents the systemic inflammatory response to select bacterial challenges in a nonhuman primate model of sepsis (33, 34, 35). However, it remains unclear why the efficacy of FXII inhibitors to prevent thromboinflammation in sepsis seems to vary depending on the mode or type of bacterial challenge. The amphipathic and anionic properties of LPS drive assembly of LPS into negatively charged aggregates in the form of micelles or vesicles in plasma, representing an ideal surface for the initiation and propagation of FXII activation in the circulation (36, 37). In 1974, Morrison and Cochrane were the first to implicate LPS as a molecular link between bacteria and FXII, when they demonstrated that the lipid A core of LPS from *Escherichia coli* binds and activates FXII in purified systems (38). They suggested that the negatively charged phosphate groups and/or the fatty acids present in the lipid A structure at physiologic pH could be responsible for the activation of FXII. Studies by Kalter and Van Dijk in the early 1980s demonstrated that the lipid A core and the variation in length and composition of the polysaccharide side chains of LPS played a key role in the generation of FXIIa (39). More recently, studies by Yang et al. demonstrated that LPS is capable of binding to HK (40). Here, we show that LPS is capable of binding to FXI and PK. Importantly, we demonstrate that the potential of LPS to bind and activate the members of the contact activation pathway is dependent on the physiochemical properties of the LPS chemotype. The findings shed new light on the role that chemotype diversity of bacteria plays in the prothrombotic and inflammatory phenotype of infections and may inform the choice of pharmacological targets to prevent thromboinflammation in sepsis.

## Results

### Activation of the contact pathway in a nonhuman primate model of *E. coli* infection

**Figure 1:**
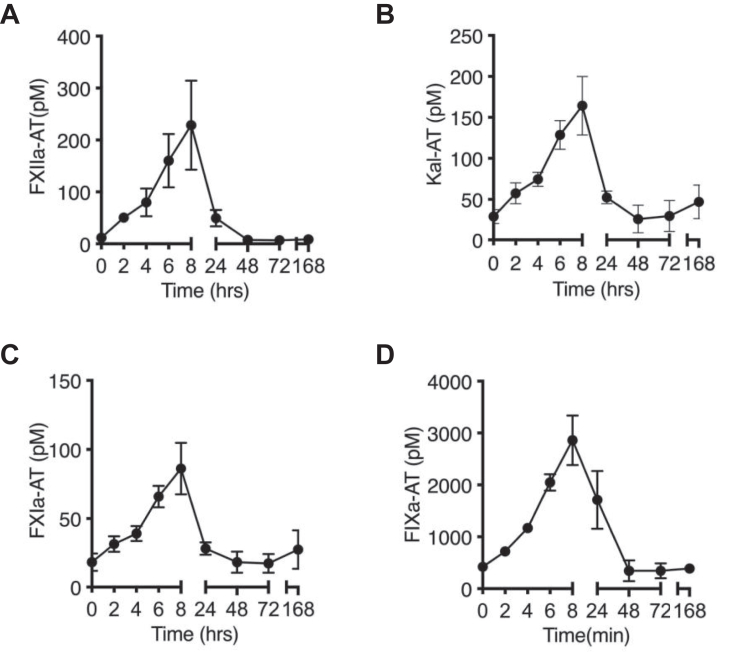
***Escherichia coli* infusion induced contact pathway activation in baboons.** Activation of contact coagulation cascade in baboons challenged with *E. coli*. Time course dynamics of complexes of (*A*) FXIIa- AT, (*B*) kallikrein-AT, (*C*) FXIa-AT, and (*D*) FIXa-AT in bacterial-challenged animals are shown.

We investigated whether the contact pathway was activated in the bloodstream of baboons challenged with a sublethal dose of *E. coli*. We observed a rapid and robust activation of coagulation FXII within the first 8 h of bacterial challenge, as measured by the extent of activated FXIIa in complex with the serpin antithrombin (FXIIa-AT; Fig. 1*A*). Consistent with this finding, we observed that the downstream members of the contact activation and intrinsic pathway, namely PK, FXI, and FIX, were likewise activated over a similar time course, as measured by kallikrein-AT, FXI-AT, and FIX-AT complexes, respectively (Fig. 1, *B*–*D*). These data provided evidence that FXII and the contact activation pathway were activated *in vivo* following infusion of *E.coli*, although it remained unclear whether this was due to a direct or indirect activation of FXII by bacterial components including LPS.

### Characterization of the physicochemical properties of LPS chemotype aggregates and coagulation factors

**Figure 2:**
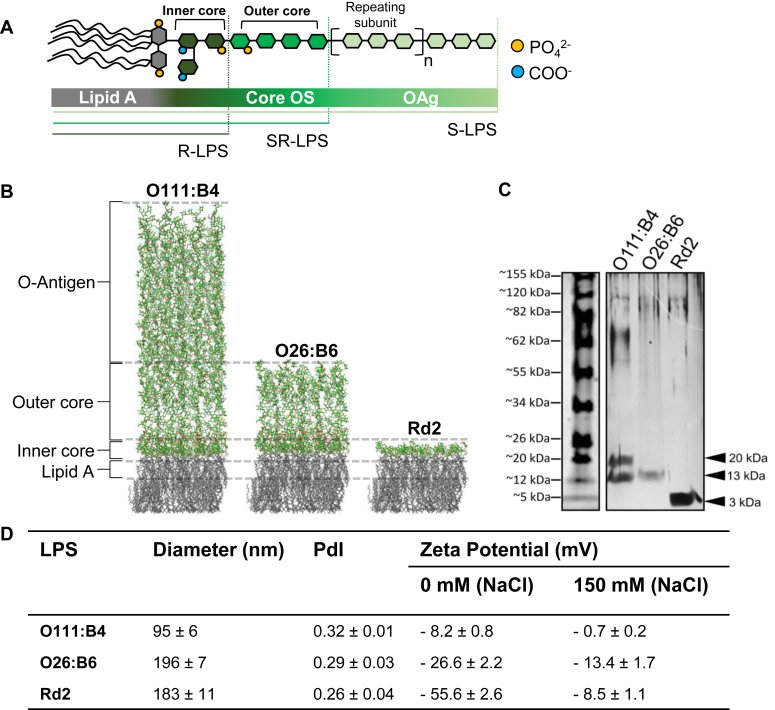
**LPS chemotypes form aggregates with distinct physicochemical characteristics.***A*, illustration of LPS molecule. LPS is composed by lipid A, core oligosaccharide (core OS; divided into an inner and an outer region), and a O-polysaccharide chain (O-Antigen; or OAg). The conserved lipid A and inner core region are highly anionic due to multiple negative charges from ${\text{PO}}_{4}^{2-}$ (*yellow* circles) and ${\text{COO}}^{-}$ (*blue* circles). LPS with a long OAg are classified as smooth strains (or S-LPS) while those lacking the OAg are considered rough strains (or R-LPS). Bacteria that express LPS chemotypes which contain only one repeating OAg subunit are classified as semi-rough strains (or SR-LPS). *B*, a model of the *Escherichia coli* outer membrane containing LPS chemotype molecules was created using CHARMM (http://charmm-gui.org/) and processed with PyMol. The lipid A moieties are shown as *gray* chains, while the inner and outer core and O-antigen are shown as *green* chains. O111:B4 (smooth or S-LPS), O26:B6 (semi-rough or SR-LPS), Rd2 (rough or R-LPS) LPS chemotypes are differentiated by the length of the O-antigen and core region. *C*, LPS chemotypes O111:B4, O26:B6, or Rd2 (50 μg/ml) were submitted to electrophoresis on a 10 to 20% polyacrylamide gel in the presence of SDS and detected by silver staining. The apparent molecular weights obtained, determined by comparison with molecular weight standards, were 20 to 100 kDa for O111:B4, 13 kDa for O26:B6, and 3 kDa for Rd2. *D*, statistical analysis and quantifications of size diameters, polydispersity index (PdI), and zeta potential (ZP) of LPS aggregates. The size distribution of LPS aggregates is shown for O111:B4 (*blue*), O26:B6 (*red*), and Rd2 (*green*), which peak with a d_h_ of 95 ± 6 nm, 196.0 ± 7, and 183 ± 11, respectively. The ZP values confirmed the expected negative surface charges of O26:B6 and Rd2, as well as the overall neutral charge of O111:B4. The aggregates were analyzed in 20 mM Hepes buffer, pH 7.4 in the absence or presence of 150 mM NaCl. The PdI of the aggregates were in general <0.3, which indicates a good stability and homogeneity.

To determine whether LPS can directly activate the contact pathway of coagulation, we studied three distinct chemotypes of LPS expressed by *E.coli*, namely the O111:B4 (S-LPS), O26:B6 (SR-LPS), Rd2 (R-LPS) chemotypes (Fig. 2*A*). As computationally modeled in Figure 2*B*, the base of the smooth LPS chemotype is composed of lipid A moieties (depicted as grey chains), followed by an inner and outer core OAg portion (shown as green chains) and capped by an extended region comprised of OAg polysaccharides. The semi-rough LPS chemotype lacks this OAg subunit, while the mutant rough LPS chemotype additionally lacks the outer core OAg. The structural differences between LPS chemotypes translated into differences in apparent molecular weight, as analyzed by SDS-PAGE (41, 42) and shown in Fig, 2C. In agreement with previously published values (43, 44, 45, 46, 47), we found that S-LPS (O111:B4) exhibited a diverse molecular weight ranging from 10 to 100 kDa, with two major bands in 10 and 20 kDa. Consistent with being comprised of a limited number of short saccharide chains, the SR-LPS (O26:B6) chemotype was detected as a single major band at 13 kDa, while the R-LPS (Rd2) LPS chemotype was detected as a ∼3 kDa band (Fig. 2*C*).

We solubilized LPS from the O111:B4, O26:B6, and Rd2 *E.coli* chemotypes in a Hepes buffer for LPS to self-assemble into aggregates. The effective radius of the aggregates was defined by dynamic light scattering (DLS). We found that each LPS chemotype produced distinct, narrowly distributed peaks, suggesting the formation of uniform aggregate populations (Fig. S1*A*). The apparent hydrodynamic diameters (d_h_) for the chemotypes O111:B4, O26:B6, and Rd2 were 95  ± 6 nm, 196  ± 7 nm, and 183  ± 11 nm, respectively (Fig. 2*D*). All three chemotypes formed aggregates with polydispersity indexes below 0.7 (Fig. 2*D*), indicative of stable and monodisperse systems (48). The three chemotypes exhibited net negative charges with distinct zeta potentials (ZPs). The addition of the monovalent cation, Na^+^, reduced or nearly neutralized the negative charges of the LPS chemotypes (Fig. 2*D*).

Last, we characterized the hydrophobicity of the LPS aggregates. Pyrene was used as a fluorescent probe, and the fluorescence intensity was collected over the 350 to 450 nm wavelength range to quantify the intensity ratio between peak I (λ = 373 nm) and peak III (λ = 384 nm) (Fig. S1*B*). The intensity ratio was lowest for the Rd2 chemotype than the O26:B6 or O111:B4 chemotypes (I373/III384 = 0.91 ± 0.005, 1.04 ± 0.04, and 1.07 ± 0.03, respectively) (Fig. S1*C*), indicative of greater hydrophobicity for the rough Rd2 chemotype than the smooth and semi-rough LPS chemotypes.

**Figure 3:**
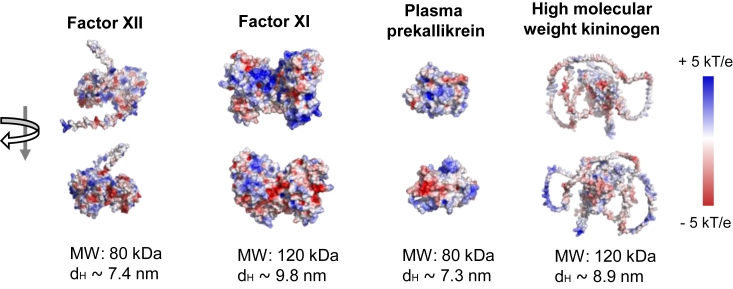
**Illustration of electrostatic potential surfaces of contact pathway proteins, calculated by the APBS module as implemented in PyMol.** The following PDB structures were used in the calculations: 5EOD (FXI) and 2ANW (PK). The PDB structure for HK and FXII was downloaded from the AlphaFold Database (entry P01042 and P00748, respectively). Additionally, the figure provides information on molecular weights and hydrodynamic diameters (H_D_).

We compared the physicochemical properties of the members of the contact pathway of coagulation FXII, FXI, PK, and HK. We modeled the electrostatic surface potentials for each protein using the Poisson–Boltzmann equation, using the PyMol visualization tool in Figure 3 (49, 50). Each coagulation factor has distinct regions of positive surface potential (pseudo-colored in blue), which may serve as potential binding sites for the anionic LPS molecules. We then characterized the physical properties of purified coagulation factors. By SDS-PAGE analysis, we showed that purified FXII, FXI, and PK zymogens were at least 94% pure and remained single bands after reduction (Fig. S2). As measured by DLS, the hydrodynamic diameters (d_h_) for FXII, FXI, PK, and HK was calculated as 7.4, 9.8, 7.3, and 8.9 nm, respectively (Fig. S3).

#### Characterization of the binding of LPS chemotypes with contact pathway proteins

We investigated whether LPS-coagulation factor binding would change the physicochemical parameters of either the LPS chemotypes or the coagulation factors themselves. Indeed, the binding of FXII to LPS caused a net increase in hydrodynamic diameter (d_h_) of 32 nm, 35 nm, and 32 nm for the chemotypes O111:B4, O26:B6, and Rd2, respectively (Fig. S4*A*). The adsorption of the FXI onto the surface of LPS caused a net increase in d_h_ of 47 nm, 51 nm, and 81 nm, while PK binding to LPS caused a net increase of 55 nm, 46 nm, and 43 nm for the chemotypes O111:B4, O26:B6, and Rd2, respectively (Fig. S4, *B* and *C*).

**Figure 4:**
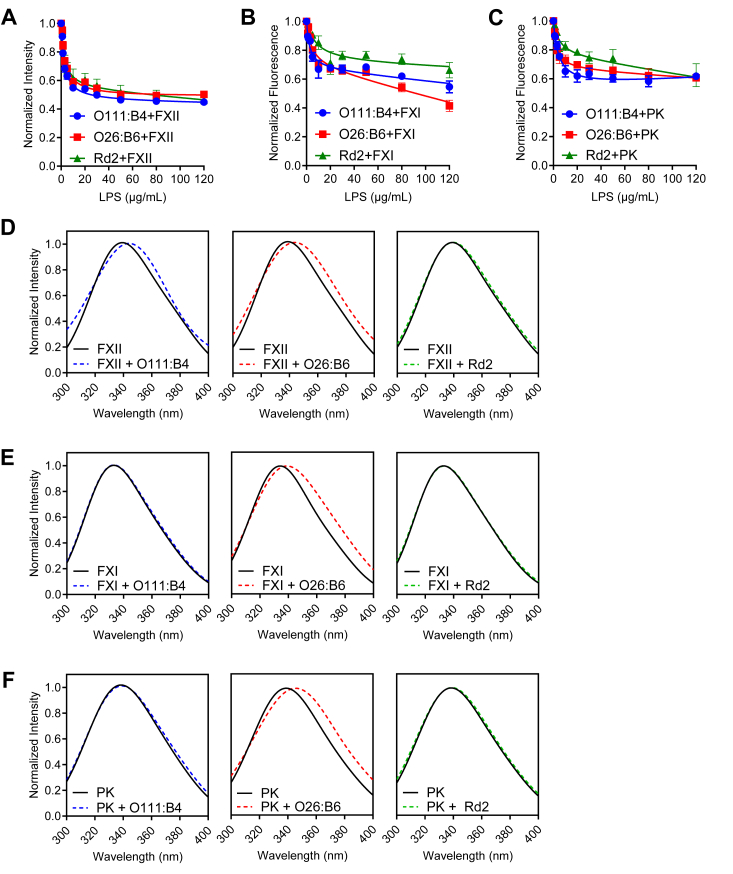
**Analysis of LPS–zymogens interactions using fluorescence spectroscopy**. The normalized tryptophan intensities at an emission of 336 nm of (*A*) FXII (0.5 μM), (*B*) PK (0.5 μM), or (*C*) FXI (0.5 μM) were plotted as a function of O111:B4, O26:B6, or Rd2 (3–120 μg/ml) in Hepes buffer solution supplemented with 150 mM NaCl. Lines are guides to the eye. Tryptophan emission spectra of (*D*) FXII (0.5 μM), (*E*) PK (0.5 μM), or (*F*) FXI (0.5 μM) in the absence or presence of LPS (120 μg/ml). Spectra were normalized from 0 to 1 for ease of comparison. The data points represent the averages of triplicate measurements, with error bars indicating ±1 SD.

Next, a fluorescence quenching technique was employed to quantify LPS-coagulation factor complex formation. This approach takes advantage of the inherent sensitivity of the fluorescent properties of tryptophan (Trp) and tyrosine (Tyr) residues to protein conformational changes (51). This technique is ideal for measuring complex formation between LPS chemotypes and the coagulation factors XII, XI, and PK, which contain 13, 10, and 11 Trp and 19, 22, and 24 Tyr residues, respectively (52, 53, 54). We observed a consistent, abrupt, and largely saturable decrease in the fluorescence emission for FXII, FXI, and PK with increasing concentrations of LPS (Fig. 4, *A*–*C*; see Fig. S5, *A*–*C* for raw fluorescence spectra), indicative of rapid and irreversible complex formation. We next quantified the spectral signature of each coagulation factor to determine whether complex formation with LPS chemotypes induced a spectral red shift, which would be indicative of a conformational change in the coagulation factor. As shown in Figure 4, *D*–*F*, the binding of O26:B6 to FXII, FXI, or PK induced a spectral red shift (Δλ) of 8.6, 7.7, and 8.2 nm, respectively, while the chemotypes O111:B4 and Rd2 only induced spectral red shifts in FXII. Taken together, our data suggest that LPS forms complexes with each member of the contact pathway and induce conformational changes in select coagulation factors depending on the LPS chemotype.

#### Effect of LPS on FXII, FXI, and PK activation

**Figure 5:**
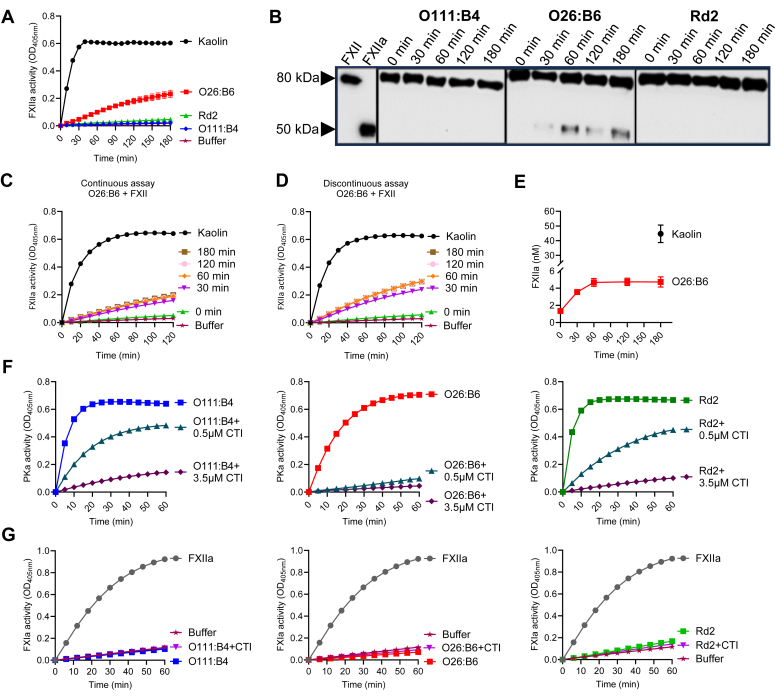
**Effects of LPS aggregates on zymogens activation.***A*, FXII (100 nM) was incubated with O111:B4, O26:B6, and Rd2 (50 μg/ml) for 2 h. Continuous formation of amidolytic activity was measured using the substrate S-2302 (300 μM) and monitored at 405 nm. Kaolin (5 μg/ml) was used as a positive control and buffer as a vehicle control. *B*, O26:B6-induced generation of FXIIa analyzed by immunblotting. FXII, 100 nM; LPS, 25 μg/ml. FXII (80 kDa) and FXIIa (50 kDa heavy chain) were used as control. *C*, assessment of FXII autoactivation in a continuous assay with O26:B6. FXII (100 nM) and O26:B6 (25 μg/ml) were incubated from 0 to 180 min, followed by the measurement of FXII amidolytic activities in a continuous reaction with S-2302 (300 μM). *D*, assessment of FXII autoactivation in a discontinuous assay with O26:B6. Timed aliquots from an FXII and O26:B6 mixture were quenched with 0.1 mg/ml polybrene, and FXII amidolytic activities were subsequently measured. *E*, apparent concentration of FXIIa generated at each quenched time point in the discontinuous assay. Concentrations were calculated from the initial velocities in (*D*) with reference to a standard curve. Kaolin promotes the formation of ∼50 nM FXIIa (*black sphere*). Data points are the averages of triplicate measurements. *F*, autoactivation of PK in the presence of LPS and HK appears to be due to contamination of HK with traces of FXII(a). The apparent autoactivation was inhibited by CTI. PKa levels were measured as a function of time after incubating PK (100 nM) with HK (120 nM) and LPS aggregates (25 μg/ml) in the presence of 0, 0.5, or 2.5 μM CTI, and PK amidolytic activities were subsequently measured using the substrate S-2302 (300 μM) at 405 nm. *G*, assessment of autoactivation of FXI in the presence of LPS. FXI (50 nM) with HK (120 nM) and LPS aggregates (25 μg/ml) were mixed, and FXI amidolytic activities were subsequently measured using the substrate S-2366 (300 μM) at 405 nm. Data are expressed as mean ± SD of three independent experiments.

We studied the consequences of LPS-coagulation factor complex formation on coagulation factor activation. We assessed autoactivation of FXII by measuring changes in amidolytic activity in a continuous assay after a 2-h incubation. While less than observed in the presence of kaolin, a powerful inducer of FXII autoactivation, we observed an increase in amidolytic activity for FXII in the presence of LPS chemotype O26:B6 (Fig. 5*A*). Formation of the 50 kDa heavy chain of FXIIa was observed in Western blots of reactions with O26:B6, but not the O111:B4 or Rd2 chemotypes (Fig. 5*B*).

We studied the potential of the O26:B6 chemotype to directly activate FXII by assessing FXIIa activity as a function of incubation time with LPS in a continuous assay, where reactions are ongoing while amidolytic activity is measured, and a discontinuous assay, where aliquots from reactions are tested at specific time points (55). We detected FXIIa generation by O26:B6 by both methods (Fig. 5, *C* and *D*). When the concentration of FXIIa at each time point was measured in the discontinuous assay, ∼5 nM FXIIa was generated by O26:B6 within the first 60 min, reflective of the conversion of ∼5% of the total FXII available in the assay from the zymogen form to an enzyme. As a comparator, ∼50% of FXII was converted from the zymogen form to generate ∼50 nM FXIIa in the presence of the highly charged silica tetrahedral kaolin (Fig. 5*E*). Moreover, the rate of FXIIa generation by the O26:B6 and Rd2 chemotypes were highly sensitive to the ionic strength of the buffer (Fig. S6). The strong dependence on the concentration of NaCl reflects the ionic nature of the interaction between FXII and LPS.

We tested the capacity of LPS to induce autoactivation of PK or FXI, as both zymogens are known to autoactivate in the presence of polyanions (56). We first studied whether incubation of PK, HK, and LPS together for 2 h resulted in the generation of PKa. We observed robust PKa generation in the presence of all three LPS chemotypes (Fig. 5*F*). Activation required addition of HK, as no measurable PKa was detected in the absence of HK (data not determine whether this was due in part to contaminating FXII that might be present in plasma-derived HK (57); we repeated these experiments in the presence of the FXIIa inhibitor, corn trypsin inhibitor (CTI). Indeed, the level of PKa generated by the LPS chemotypes was reduced and, in the case of the O26:B6 strain, nearly eliminated, in the presence of increasing concentrations of CTI (Fig. 5*F*), suggesting as a cautionary note that amidolytic activity data derived using plasma-derived HK might be in part due to contaminating FXII (57). This was not the case for FXI, as while the degree of FXIa generated in the presence of the LPS chemotypes was lower than that observed in reactions in which FXIIa was added; the amidolytic activity of FXIa generated by autoactivation with LPS was insensitive to CTI (Fig. 5*G*).

### Effect of LPS on FXIIa, FXIa, and PKa activity

**Figure 6:**
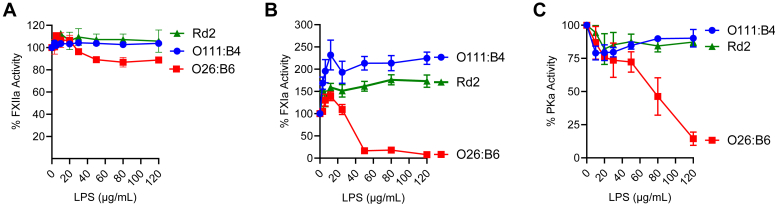
**Effects of LPS on FXIIa, PKa, and FXIa amidolytic activities.** (*A*) FXIIa (50 nM), (*B*) PKa (50 nM), or (*C*) FXIa (50 nM) was incubated with LPS (0–120 μg/ml), and amidolytic activity was measured using the substrate S-2302 (300 μM) for FXIIa or PKa and S-2366 (300 μM) for FXIa. The LPS showed distinct abilities to regulate the catalytic function of the proteases. Data are expressed as mean ± SD of three independent experiments.

We examined the effects of LPS chemotypes on the catalytic properties of the enzymes FXIIa, FXIa, or PKa toward small chromogenic substrates. FXIIa cleavage of chromogenic substrate was unaffected by O111:B4 or Rd2, while a reduction in amidolytic activity approaching 20% was observed at the highest concentrations of O26:B6 (Fig. 6*A*). Enhanced enzymatic activity was observed for FXIa in the presence of increasing concentrations of O111:B4 or Rd2 (Fig. 6*B*), while these two chemotypes produced the opposite and inhibitory effect on the amidolytic activity of PKa (Fig. 6*C*). The O26:B6 chemotype dose-dependently inhibited the amidolytic activity of PKa (Fig. 6*C*), while a biphasic response of reduced activity at high concentrations compared to enhanced activity at low concentrations was observed for the amidolytic activity of FXIa (Fig. 6*B*). Taken together, these data suggest that the functional effects of LPS chemotypes on the activated form of coagulation factors are heterogeneous and vary by species, perhaps inducing allosteric changes in the catalytic domains of the contact factors.

#### FXII–LPS complexes promotes FXI and PK reciprocal activation

**Figure 7:**
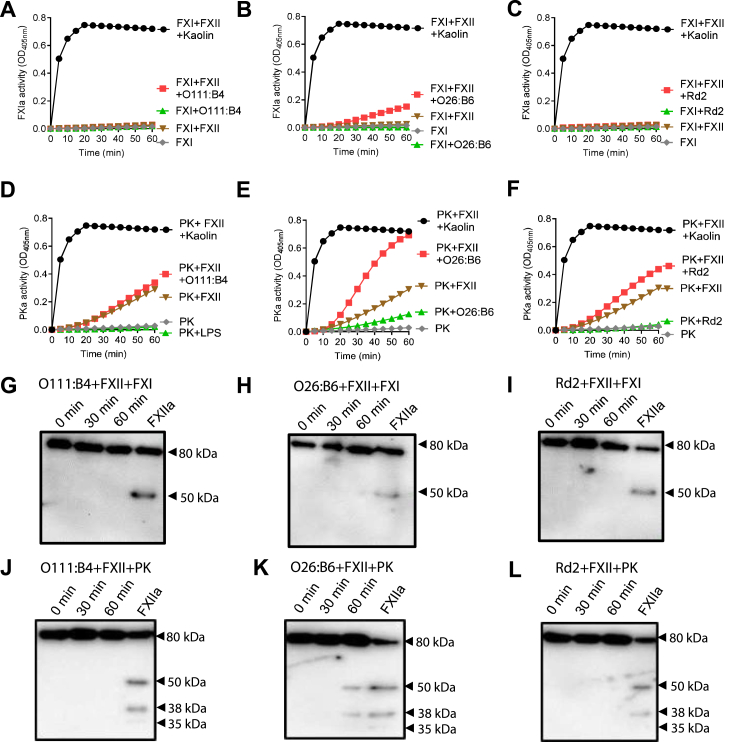
**The complex FXIIa–LPS presents catalytic activity towards FXI and PK.** FXI (30 nM) was incubated with FXII (5 nM), with (*A*) O111:B4, (*B*) O26:B6, and (*C*) Rd2 (25 μg/ml) or with both in the presence of S2302 (300 μM) and the reaction was subsequently monitored at 405 nm. Samples from 0, 30, and 60 min time point were separated under reducing conditions and immunoblotted with an antibody against FXI in the presence of (*G*) O111:B4, (*H*) O26:B6, or (*I*) Rd2. PK (50 nM) was incubated with FXII (5 nM), with (*D*) O111:B4, (*E*) O26:B6, and (*F*) Rd2 (25 μg/ml) or with both in the presence of S2302 (300 μM) and the reaction was subsequently monitored at 405 nm. Samples from 0, 30, and 60 min time point were separated under reducing conditions and immunoblotted with an antibody against PK in the presence of (*J*) O111:B4, (*K*) O26:B6, or (*L*) Rd2. Data are expressed as mean ± SD of three independent experiments.

We tested whether the formation of a complex with LPS changed the enzymatic activity of coagulation factors. We measured amidolytic activity in a discontinuous assay as a way to first study whether FXII in complex with LPS exhibited protease activity toward the zymogens PK or FXI. We first confirmed that incubation of the zymogen forms of FXI and FXII alone was insufficient to generate substantial levels of FXIa as measured by cleavage of the chromogenic substrate S2366 (Fig. 7, *A*–*C*), while the incubation of PK with FXII was sufficient to generate PKa activity as measured by cleavage of the chromogenic substrate S2302 (Fig. 7, *D*–*F*). Of note, the PKa measurements were without exogenously added HK and performed in the presence of CTI to ensure specificity, as the chromogenic substrate used to measure PKa activity can be cleaved by both PKa and FXIIa.

We found that only the O26:B6 chemotype in complex with FXII enhanced, albeit only slightly, the activation of FXI (Fig. 7, *A*–*C*). Along these lines, the O26:B6 chemotype in complex with FXII was capable of robustly enhancing the activation of PK to PKa (Fig. 7*E*). Interestingly, a slight increase in the rate of PK activation was observed for FXII when in complex with the LPS chemotype Rd2 (Fig. 7*F*). These results were further analyzed by Western blot. Although a slight increase in the amidolytic activity of the substrate S-2366, specific for FXIa, was observed when FXI was in contact with the FXII–O26:B6 complex, no band indicative of FXIa generation (50 kDa) was detected by Western blot (Fig. 7*H*). This discrepancy could be attributed to the higher sensitivity of the enzymatic assay, which can detect the enzyme at low concentrations. O111:B4 (Fig. 7*G*) and Rd2 (Fig. 7*I*) also did not generate FXIa. The O26:B6 chemotype was capable of complexing with FXII to catalyze the cleavage of PK to generate PKa, resulting in the loss of the 80 kDa band of PK and appearance of the 50, 38, and 35 kDa bands corresponding to PKa (Fig. 7*K*). Although the FXII–Rd2 complex promoted a slight generation of PKa, as indicated by amidolytic activity, no visible band corresponding to PKa was detected by Western blot, possibly due to the low levels of PKa generation being insufficient for detection by this method (Fig. 7*L*). The chemotype O111:B4 failed in generating PKa (Fig. 7*J*).

#### LPS chemotypes display distinct biological activity in the form of aggregates or as monomers

**Figure 8:**
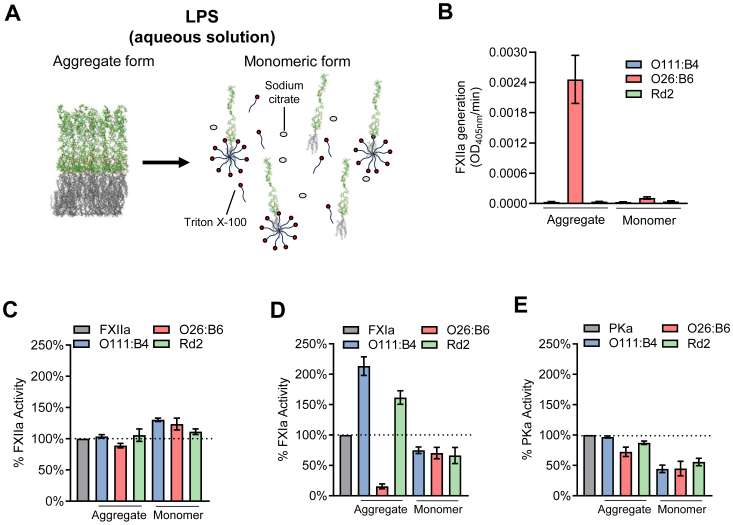
**Effects of “masked” monomeric LPS on contact pathway proteases.***A*, schematic representation of LPS masking. In aqueous solution, LPS naturally forms aggregates in the form of micelles or vesicles. Addition of citrate (chelator) weakens the aggregate structure by removing divalent cations. Surfactants like Triton X-100 then intercalate into the aggregates. Eventually, the aggregates completely disperse into monomers. *B*, FXIIa generation in the presence of LPS aggregates or monomers. LPS (50 μg/ml) in the form of aggregates or monomers were incubated with FXII (100 nM) for 2 h followed by the addition of S-2302 (300 μM). LPS in their aggregate form are more potent surfaces to stimulate FXII autoactivation than monomers. The effects on the proteases (*C*) FXIIa, (*D*) PKa, and (*E*) FXIa in the presence of aggregates or monomers were evaluated. The *dashed line* indicates the point of maximum enzyme activity (100%). Data are expressed as mean ± SD of three independent experiments.

It has been reported that LPS aggregates (micelles or vesicles) represent the biologically active form (58). We evaluated the impact of LPS as aggregates or in monomeric form on the activation and activity of contact pathway factors. As depicted in Figure 8*A*, we used chelators to “mask” the LPS chemotypes by weakening the structure of the aggregates, enabling detergents to intercalate and promote the formation of LPS monomers (59). Our results show that the potential of LPS O26:B6 to activate FXII was abrogated in the monomeric form (Fig. 8*B*). Chemotypes O111:B4 or Rd2 were incapable of activating FXII in the aggregated or monomeric form. This suggests that the LPS aggregates represent the minimal biological unit for promoting FXII autoactivation.

We next examined the effects of LPS aggregates and monomers on FXIIa, PKa, or FXIa amidolytic activity. We observed that the monomeric form of all three LPS chemotypes promoted the amidolytic activity of FXIIa, while reducing the catalytic activity of FXIa and PKa (Fig. 8, *C*–*E*). An interesting effect was observed for the effects of LPS aggregates on the catalytic activity of FXIa, wherein the O111:B4 and Rd2 chemotypes increased the rate of the activity of FXIa while the O26:B6 chemotype all but eliminated the catalytic activity of FXIa (Fig. 8*D*).

#### LPS chemotypes promote coagulation in plasma

**Figure 9:**
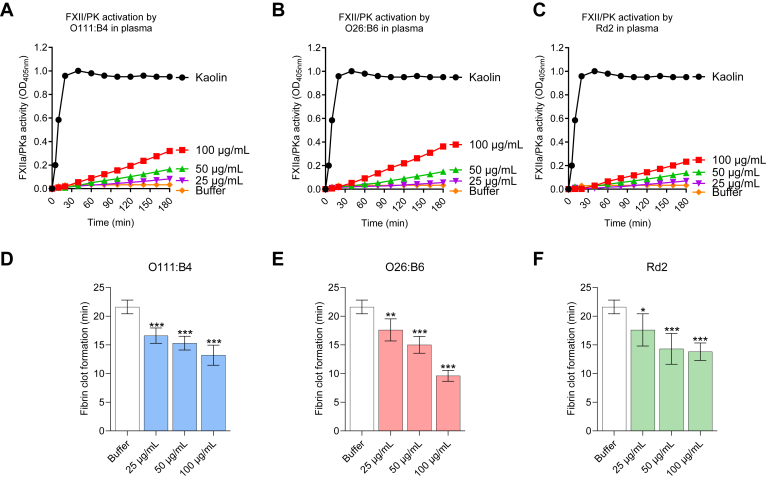
**Detection of FXII/PK activation during contact activation in plasma by LPS.** Contact activation was triggered by the incubation of (*A*) O111:B4, (*B*) O26:B6, or (*C*) Rd2 (25, 50, or 100 μg/ml) in citrated plasma for 30 min at 37 °C, followed by the measurement of FXII/PK amidolytic activity using the substrate S-2302 (300 μM) and monitored at 405 nm. Clot times of human normal plasma by (*D*) O111:B4, (*E*) O26:B6, or (*F*) Rd2 (25, 50, or 100 μg/ml) after incubation for 5 min. After recalcification, time to clot formation was assayed by turbidity measurements at 405 nm. Kaolin (5 μg/ml) was used as a positive control and buffer as a vehicle control. Error bars are the SD of three independent experiments performed in technical triplicate. Results were analyzed by one-way ANOVA; ∗*p* < 0.05, ∗∗*p* < 0.001, ∗∗∗*p* < 0.0001 with respect to buffer control.

We examined whether the LPS chemotypes can activate the contact pathway of coagulation in plasma. First, we validated the colloidal stability of LPS aggregates in plasma, where we observed a mixed population of large and small aggregates (Fig. S7*A*). This might reflect the disaggregating effects caused by proteins such as albumin, alongside the formation of a biomolecular corona (60, 61, 62). Furthermore, ZP measurements indicate that the composition of the biomolecular corona remains relatively stable as plasma concentrations increase (Fig. S7*B*). Generally, the adsorption of plasma proteins onto negatively charged nanoparticles results in changes to the ZP, which depend on both the quantity and type of proteins that are bound (63). In buffer solution, the LPS chemotypes O111:B4, O26:B6, and Rd2 displayed a ZP of −8.2, −26.6, and −55.6 mV, respectively (Fig. 2*D*). Following incubation with 1% human plasma, the ZP markedly increases to around −7.1, −10.2, and −11.1 mV, respectively, implying that a relatively complete corona layer is established even with low concentrations of plasma. Increasing the plasma concentration to 5% and 10% resulted in minimal changes in the ZP, which implies that the qualitative composition of the biomolecular corona does not vary significantly (Fig. S7*B*). We then validated the potential of the LPS chemotypes O111:B4, O26:B4, and Rd2 to promote FXII activation in plasma. We performed these experiments in the absence of calcium, which is not required for the contact pathway but is required for the downstream steps of thrombin generation and fibrin formation. We found that all three LPS chemotypes were able to cause an LPS-concentration dependent increase in FXIIa/PKa amidolytic activity in plasma in the absence of calcium as measured by the cleavage of the chromogenic substrate S-2302 (Fig. 9, *A*–*C*). When calcium was added to permit downstream thrombin generation, we observed that all three LPS chemotypes were able to promote clotting and thus reduce the time to generate fibrin (Fig. 9, *D*–*F*).

## Discussion

Components of bacterial cell walls are known to interact with components of the contact pathway. Bacterial LPSs promote reciprocal activation of FXII and PK, forming FXIIa and PKa (38, 39). In the present study, we took an important step toward understanding the physical properties of distinct LPS chemotype aggregates and the role that their structural and surface properties contribute to regulating the binding and activation of contact pathway zymogens.

The physiochemical signature of biological surfaces plays a critical role in triggering contact activation. Prior investigations have shown that the ZP of different *E. coli* strains range from −3.5 to −49 mV (64). The negative charge of Gram-negative bacteria is attributed to the presence of negatively charged lipids and LPS molecules in their outer membrane that are likely responsible for triggering coagulation *via* the contact pathway. In a conserved manner, the activation of FXII on bacterial surfaces as well as procoagulant microvesicles has been linked to thrombin generation in patients with septic disseminated intravascular coagulation (65). Here, we demonstrated that *E. coli* can trigger coagulation through the activation of contact pathway proteases. A mechanistic role for the bacterial wall component LPS in contact activation has been described. While the physicochemical properties of LPS are known to be a key determinant in endotoxic activity, their importance to FXII activation is relatively unexplored. It has been shown that the aggregate form of LPS is required for biological activity, while monomeric LPS is inactive (58). Based on the assumption that LPS aggregates in plasma, the biophysical and chemical composition indicates a procoagulant potential, unless its negatively charged sites are shielded by polysaccharide chains. We tested the hypothesis that the physicochemical properties of specific LPS isoforms predict the potential to activate the contact pathway.

We found that the smooth chemotype O111:B4 produced homogeneous and neutrally charged aggregates, unlike the semi-rough (O26:B6) and rough (Rd2) chemotypes, which exhibited highly negatively charged surfaces. The ZP magnitude provides valuable insight into the stability of colloidal aggregates. A higher potential magnitude results in stronger electrostatic repulsion, contributing to enhanced stability (66). Therefore, our data indicate that the repulsion forces of Rd2 aggregates are greater than O26:B6 and O111:B4 (R-LPS > SR-LPS > S-LPS). We also confirmed the role of monovalent cations in adsorbing and neutralizing LPS surface charge (67, 68). Despite the physical differences among the chemotypes, when qualitatively measured by fluorescence spectroscopy, we found that FXII, FXI, and PK bound to the surface of O111:B4, O26:B6, and Rd2. However, the multisite interactions make it challenging to accurately quantify the fluorescence quench data. In fact, FXII is a molecule that may have multiple surface-binding domains and a hydrodynamic diameter of approximately 7.4 nm, smaller than that of LPS (∼100–200 nm). This suggests that a single LPS aggregate could accommodate multiple proteins at a time. FXII (pI ∼ 6.3) is composed of a noncatalytic and a catalytic domain. The noncatalytic portion of the protein, referred to as the heavy chain, has a net positive charge which promotes FXII surface-binding, and it might be the principal site of interaction with LPS. On the other hand, the catalytic domain, also referred to as the light chain, is the negatively charged portion of the FXII which will be accessible to interact with substrates upon binding to LPS (69, 70). In similar fashion to FXII, FXI (D_H_ ∼ 9.8 nm) and PK (D_H_ ∼ 7.3 nm) also exhibit cationic surface regions to which LPS could bind (53, 54). The findings demonstrate that the binding of the coagulation factors to the aggregate surface is due to electrostatic interactions between the cationic side chains on the zymogen’s surface and the anionic lipid core portion of the LPS.

When examining fluorescence spectra of complex formation between LPS with FXII, FXI, or PK, we observed that the binding induced a conformational change in the global structure of the zymogens, with a more substantial change when interacting with the chemotype O26:B6, which is the only one capable of generating FXIIa. It is noteworthy that the generation of FXIIa was dependent on the chemical environment and composition of LPS (67). The smooth O111:B4 chemotype was not capable of activating FXII regardless of the ionic strength. At low ionic strength, the chemotype O26:B6 presented a negatively charged surface, promoting substantial activation of FXII. Although the ZP of O26:B6 aggregates increased at physiological salt conditions (150 mM NaCl), the aggregates remained negatively charged with the O26:B6–FXIIa complex exhibiting significant amidolytic activity. Chemotype Rd2, on the other hand, exhibited a highly negative surface charge at low NaCl concentrations and, thus, a strong potential for FXII activation under those conditions. However, with the increase in NaCl concentration, the Rd2 chemotype aggregate was neutralized leading to reduced formation of amidolytic activity. This result can be explained by the fact that the saccharide moiety of LPS hydrodynamically and sterically shields the negatively charged core of the aggregate. This property, however, is dependent on the saccharide chain length and the chemical environment. We observed that the long saccharide chain groups in S-LPS would likely lead to minimal electrostatic contributions of the negatively charged LPS core to the free energy of interaction with positively charged sites on FXII both in the absence or presence of Na^+^. On the other hand, SR-LPS has a short oligosaccharide chain that is not dense enough to completely shield the core of the aggregate, resulting in partial neutralization of the negatively charged phosphate groups with increasing concentration of monovalent cations. In the R-LPS chemotype, the phosphate groups of the lipid A region are more exposed to the surrounding environment, and as Na + concentration increases, a decrease in FXII activation is observed (6).

Previous research indicates that the binding of FXII with specific anionic surfaces may modulate the activation of PK and FXI. The interaction between PK and FXII can result in mutual activation even in the absence of a surface. While the presence of a surface may accelerate these reactions, they are not strictly surface-dependent (71). In contrast, the activation of FXI by FXIIa, thrombin, or *via* autoactivation is surface-dependent and requires the presence of a surface for efficient activation (29). Our study revealed that the LPS–FXII complex facilitated the activation of PK and FXI based on the specific chemotype. While the O26:B6– or Rd2–FXII complex served as an activating surface for PK, only the O26:B6–FXII showed the capacity to activate FXI. Notably, O111:B4–LPS appeared to have limited capability to promote zymogen activation. Additionally, studies have shown that certain polyanions can trigger coagulation through direct activation of FXI or PK, even in the absence of FXIIa. Research has shown that FXI and PK can be activated through polyanions independently of FXII. Polyanions like polyphosphate enhance FXI autoactivation and activation by thrombin, bypassing the need for FXII. Additionally, in some contexts, PK is involved in thrombin generation without FXII involvement, potentially through other pathways such as direct activation by thrombin or other polyanion-mediated mechanisms. These findings suggest alternative activation mechanisms for FXI and PK, independent of FXII (72, 73, 74). Nevertheless, we observed that the LPS aggregates did not display an appropriate biological surface to induce PK or FXI autoactivation.

It has been reported that certain pathogen-derived molecules can regulate the activity of the contact pathway proteases. Popescu *et al.* showed that anthrax-derived peptidoglycan (a large polymer composed of polysaccharide and peptide) interacts with and modulates the activation of contact pathway zymogens. They reported that, although peptidoglycan does not directly activate FXII or PK, the polymer moderately enhanced the catalytic activity of FXIIa and PKa (75). Galochkina *et al.* developed a mathematical model to investigate the activation of the contact pathway by LPS. Using computational models, they suggested the existence of an optimal LPS concentration for the contact pathway activation where low concentrations promoted a limited rate of activation, and high concentrations caused an apparent inhibition (76). In this study, we observed that the LPS can regulate FXIa and PKa catalytic activities. While the chemotypes O111:B4 and Rd2 did not substantially affect the activity of PKa, the LPS O26:B6 acted as a dose-dependent inhibitor of the kallikrein amidolytic activity. On the other hand, the affinity of FXIa for the substrate is enhanced in the presence of O111:B4 and Rd2. However, the bell-shaped dose-response curve for the FXIa catalytic activity in the presence of O26:B6 may suggest an allosteric effect, acting as both activator and inhibitor. The activity of FXIIa was minimally affected only by O26:B6 chemotype. Interestingly, the LPS O26:B6 is a component of the membrane of O26 strains, one of the most virulent strains of *E. coli* (77, 78). A potentially fatal condition termed hemolytic uremic syndrome (HUS) is caused in part by the Shiga toxin-producing *E. coli* (STEC) O26 strains. This syndrome is characterized by increased coagulation activation, microthrombi formation, hemolytic anemia, thrombocytopenia, and organ damage (79, 80). Herein, we demonstrated that LPS from O26 strains can activate the contact pathway *via* direct activation of FXII and regulate FXIa and PKa activities, which, in conjunction with Shiga toxin, may play a role in the development of hemolytic uremic syndrome.

**Figure 10:**
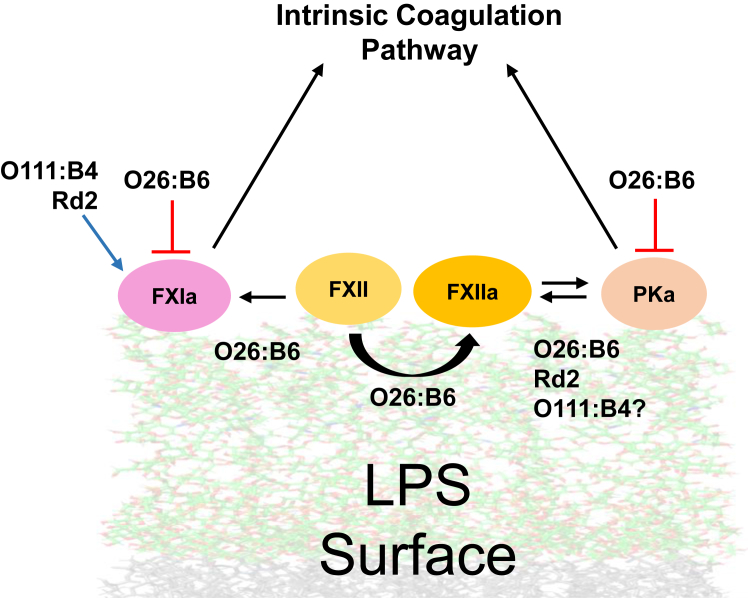
**Simplified schematic illustration of the contact pathway activation by LPS**. Although the zymogens FXII, FXI, and PK bind to the LPS surface, the nature of their interactions and influence differ according to the chemotype. Only FXII autoactivates upon O26:B6 chemotype LPS surface contact, generating FXIIa. The FXII–O26:B6 complex activates both PK and FXI, whereas the FXII–Rd2 activates only PK. The LPS chemotypes also uniquely regulate protease catalytic activities. O26:B6 acts as a regulator for PKa and FXIa showing a dose-dependent reduction of amidolytic activity (*red arrows*), while Rd2 and O111:B4 boost the catalytic activity of FXIa (*blue arrow*).

The findings are the first to demonstrate the physical mechanism of interaction, activation, and regulation of FXII, FXI, and PK by LPS in purified systems. Our results suggest that LPS aggregates activate the contact pathway through mechanisms influenced by the physicochemical properties of the aggregates. The chemical composition of LPS plays a key role in activating contact factors, as the polysaccharide chains may shield the negatively charged regions of the molecule. Several polysaccharides, including cellulose, chitosan, alginate, and hyaluronic acid, have been recognized for their hemostatic properties (81). Given that sugar-containing molecules like LPS from bacterial cell envelopes interact with proteins like Toll-like receptors on the plasma membrane—triggering the initial steps of immune response and inflammation—it is conceivable that the sugar chemistry of LPS chemotypes may contribute to the differential effects we observed for the contact proteases activities (82, 83). Nonetheless, we found that while contact pathway factors can interact with a number of LPS chemotypes under physiological conditions, the interaction of FXII, FXI, and PK with the semi-rough aggregate O26:B6 is the most notably complex capable of promoting the enzymatic regulation. Electrostatic forces are considered fundamental and required for the activation of FXII by charged surfaces and, by extension, initiation of contact activation pathway of the coagulation cascade. The data provide novel insights into the role that the blood microenvironment and the physicochemical properties of select LPS aggregates participate in regulating the nonspecific interactions and enzymatic regulation of FXII, FXI, and PK with bacterial surfaces (summarized in Fig. 10). The complex formation between FXII and LPS released from bacteria during infections may provide the initiating event to stimulate the activation of FXI and PK, subsequently leading to the generation of FXIIa by kallikrein, promoting coagulation and inflammation.

## Experimental procedures

### The baboon model for *E. coli* sepsis

EDTA anticoagulated plasma samples from a control group in a historical experiment (84) were used to gauge the activation of the contact pathway. This research adhered to ethical guidelines outlined in the Animal Welfare Act, the Guide for the Care and Use of Laboratory Animals (85), and the NIH Office of Laboratory Animal Welfare. Approval for the study protocol was obtained from the Institutional Animal Care and Use Committees of both the Oklahoma Medical Research Foundation and the University of Oklahoma Health Science Center.

The study included four healthy *Papio anubis* baboons, both males and females, aged 3 to 4 years, weighing between 6 and 10 kgs, with hemoglobin levels exceeding 10 gm/dl and white blood cell counts lower than 12,000 per microliter of blood.

As previously detailed (84), the experimental procedure entailed inducing sepsis by intravenously infusing 1 to 2 × 10^10^ *E. coli* O86a:K61 (ATCC 33985) over a period of 2 h. Blood samples and physiological data were collected at various intervals: before the *E. coli* infusion (T0), then at 2, 4, 6, and 8 h, and subsequently at 24, 48, 72, and 168 h following the bacterial challenge. Plasma samples were preserved at −80 °C for analysis.

### ELISA assays

Factor XIIa–antithrombin (FXIIa-AT), kallikrein (Kal)–AT, FXIa–AT, and FIXa–AT complexes were quantified as previously described (34). Standards were prepared by incubating FXIIa or Kallikrein or FXIa or FIXa (1 μM) with antithrombin (5 μM), EDTA (1 mM), and heparin (5 Unit/ml) for 2 h at 37 °C. Standards were defined as 1 μM of protease–antithrombin complex (86).

### Physicochemical characterization of LPS and coagulation factors

#### Sodium-dodecyl sulphate polyacrylamide gel electrophoresis

The apparent molecular weights of commercially available LPS chemotype O111:B4 and O26:B6 from *E. coli* and Rd2 mutant chemotype LPS from F583 *E. coli* (Sigma, #L2630, #L8274, #L6893) were determined by SDS-PAGE with a 12% acrylamide gel containing 0.5% SDS. LPS samples of 50 μg/ml were prepared in endotoxin-free ultra-pure water (Sigma) and applied to the gel. The LPS bands were visualized by silver staining technique (Pierce Silver Staining Kit, Thermo Fisher Scientific).

#### LPS aggregates preparation

1 mg/ml of the LPS chemotypes O111:B4, O26:B6, or Rd2 (Sigma) were solubilized in 20 mM Hepes buffer, 150 mM NaCl, pH 7.4 and extensively vortexed for 10 min, sonicated for 30 min in a water bath at 60 °C, and subjected to several temperature cycles between 4 and 60 °C. Finally, the LPS aggregates suspension was incubated at 4 °C for at least 12 h before experiments (12, 87).

#### DLS and polydispersity index

The size distribution of LPS aggregates were determined by DLS using a Malvern Zetasizer Nano ZS device (ZS-90/Malvern) equipped with a 4-mW He–Ne laser (λ = 633 nm) and with a backscattering detection at 173°. LPS aggregates were diluted to 50 μg/ml of O111:B4 or O26:B6 (Corresponding to 2.5 or 3.8 μM), respectively. Considering that the molecular weight is 20 kDa for O111:B4, 13 kDa for O26:B6), and 3 kDa for Rd2 (Corresponding to 5 μM) in 20 mM Hepes buffer, pH 7.4, the samples were poured into cuvettes for analysis at a constant temperature of 25 °C. Intensity autocorrelation functions were analyzed using the CONTIN method (88, 89), supplied with the instrument, yielding a distribution of diffusion coefficients (D). The measured D is used for the calculation of the hydrodynamic diameter (d_h_) through the Stokes-Einstein relationship. A set of 10 measurements (∼100 runs each) for the LPS aggregates were conducted to calculate the d_h_ value and the polydispersity index (12).

#### Zeta potential

The variation of ZP of the LPS aggregates was determined at 25 °C from the mean of 10 measurements (∼50 runs each) using a Malvern Zetasizer Nano ZS device (ZS-90/Malvern). Electrophoretic mobility was calculated by laser Doppler anemometry at a scattering angle of 90°. Values of refractive and viscosity index were set at 1.330 and 0.8872 cP, respectively (90). Each concentration of O111:B4, O26:B6, or Rd2 were fixed in 50 μg/ml dissolved in 20 mM Hepes buffer, pH 7.4, in the absence and presence of 150 mM NaCl. In a separate set of experiments, the ZP of LPS was measured in 20 mM Hepes buffer in the presence of 1, 5, or 10% human plasma. The electrophoretic mobility obtained was used for the ZP calculation using the Smoluchowski approximation (91).

#### Fluorescence of pyrene probes

The hydrophobicity of LPS aggregates were characterized using pyrene, a polycyclic aromatic hydrocarbon, as a fluorescent probe for both assessing polarity and studying aggregate formation. By analyzing the fluorescence spectrum of pyrene, we can estimate the polarity of the LPS microenvironment, allowing us to obtain an index of hydrophobicity (92). The emission spectrum of pyrene is constituted by five peaks, named I, II, III, IV, and V. An increase of the intensity of peak I is observed in polar solvents, while no effect is seen on the intensity of peak III. Hence, alterations in the intensity ratio of I/III offers an understanding into the dynamic polarity of the surrounding environment of the molecular pyrene probe, thus signaling the containment of this molecule within the LPS aggregate (93). Briefly, 10 μl of pyrene (Sigma-Aldrich) solution (0.5 mg/ml in acetone) was added into a series of glass test tubes, and the solvent acetone was evaporated at room temperature for 5 h. Two milliliters of LPS in 20 mM Hepes buffer, 150 mM NaCl, pH 7.4, (final concentration, 100 μg/ml) was added into the pyrene-containing tube separately to obtain the final concentration of pyrene of 2.5 μg/ml. Upon sonication for 10 min in a water bath at 25 °C, the solutions were kept at room temperature and equilibrated for 24 h before fluorescent emission measurements with the excitation wavelength of 335 nm using a CARY Eclipse fluorescence spectrophotometer in a 10 mm × 10 mm quartz cuvette. The spectra were recorded in the 350 to 450 nm wavelength range. The excitation and emission slits were set at 5 and 2.5 nm, respectively. The hydrophobic ratio was calculated by dividing the intensity of the first fluorescence peak (peak I, λ = 373 nm) by that of the third peak (peak III, λ = 384 nm). The hydrophobicity correlates inversely with the I_373_/III_384_ ratio.

#### Electrostatic calculations of contact pathway proteins

The structures of FXI (PDB code 5EOD) and PK (PDB code 2ANW) were obtained from the Protein Data Bank (PDB) (https://www.rcsb.org). The PDB structure for HK (entry P01042) and FXII (entry P00748) was downloaded from the AlphaFold Database (https://alphafold.ebi.ac.uk) (94, 95, 96). The electrostatic surface potentials were calculated for all the proteins at pH 7.4 and 150 mM NaCl using the APBS software based on the Adaptive Poisson–Boltzmann Solver (APBS) and visualized using the PyMol visualization tool (49, 50). These resources are available from the APBS website: http://www.poissonboltzmann.org/.

### Biophysical characterization of LPS–zymogens interactions

#### Dynamic light scattering

The hydrodynamic diameter of FXII/LPS complexes were determined by DLS using a Malvern Zetasizer Nano ZS device. The samples were prepared in Hepes buffer at concentrations of 50 μg/ml LPS chemotypes and 0.5 μM FXII (Prolytix), FXI (Prolytix), or PK (Enzyme Research). The solutions were poured into cuvettes for analysis at a constant temperature of 25 °C. A set of 10 measurements (∼100 runs each) for the LPS aggregates were conducted to calculate the d_h_ value.

#### Intrinsic Trp fluorescence

The fluorescence spectroscopy experiment was performed using CARY Eclipse Fluorescence Spectrophotometer and a quartz cuvette at 25 °C. Complex formation between FXII, FXI, or PK and LPS was first analyzed by fluorescence quenching titration measurements. Intrinsic Trp Fluorescence spectra of native FXII (Prolytix), FXI (Prolytix), or PK (Enzyme Research) were recorded using 0.5 μM protein diluted in 20 mM Hepes buffer, 150 mM NaCl, pH 7.4 and the interactions with LPS were studied by titrating the protein with increasing LPS aggregates concentration up to 120 μg/ml. The intrinsic Trp was excited at a wavelength of 280 nm and the emission monitored between 300 to 400 nm and with a slit width of 5 nm. The internal filter effect from LPS was accounted for by titrating a solution of the amino acid tryptophan (Thermo Fisher Scientific) with identical concentrations of LPS. Corrected quenching curves for the native proteins were generated by dividing the uncorrected data by the Trp reference curve. Emission intensities at 336 nm were recorded ∼1 min after each titration point and plotted. A skewed Gaussian function was applied for the determination of the wavelengths of maximum emission (97).

### LPS–contact pathway enzymatic assays

#### Chromogenic substrate experiments

Enzymatic activity of the contact pathway proteases can be analyzed by the amidolytic activity of synthetic substrates. These substrates are typically composed of small peptides linked to a chromogenic or fluorogenic group. Substrate cleavage results in changes to optical properties, which can be observed through light absorption or fluorescence spectroscopy (98). Contact pathway activity in a purified system or plasma is commonly tracked with the tripeptide substrate S-2302 (H-D-Pro-Phe-Arg-pNA) for detecting the activity of FXIIa and PKa and the tripeptide substrate S-2366 (Glu-Pro-Arg-*p*-NA·HCl) for detecting the activity of FXIa (99, 100). Notably, as S-2302 can be cleaved by both FXIIa and PKa, the use of specific inhibitors—such as CTI for FXIIa and soybean trypsin inhibitor for PKa—is essential to prevent their respective contributions to substrate cleavage during contact activation in plasma or purified systems (99).

#### FXII autoactivation

All chromogenic assays were performed in 96 well microtiter plate and absorbance were determined at 405 nm in a Tecan microplate reader. Prior to performing chromogenic assays, microtiter plate wells were blocked in 20 mM Hepes buffer containing 1% PEG-20,000 for 1 h. To assess FXII (Prolytix) activation, 100 nM FXII was dissolved in 20 mM Hepes buffer, pH 7.4, supplemented with 150 mM NaCl and preincubated with 25 μg/ml of O111:B4, O26:B6, or Rd2 aggregates for 2 h at 37 °C. Three hundred micromolars of the chromogenic substrate S-2302 (Chromogenix) for FXIIa was added and the amidolytic activity were then recorded using a plate reader. FXIIa generation was monitored for 180 min at 37 °C. At various times (0, 30, 60, 120, and 180 min), aliquots were removed, treated with loading buffer containing 0.2 mM DTT, and heated at 95 °C for 5 min. The samples were loaded onto a 10% SDS-PAGE gel and after electrophoresis transferred to polyvinylidene difluoride membranes. The membranes were blocked for 60 min with 5% nonfat milk in TBST (50 mM Tris, 150 mM NaCl, 0.1% Tween 20, pH 7.5), followed by incubating with anti-FXII monoclonal primary antibody (Santa Cruz Biotech) in 5% bovine serum albumin in TBST overnight at 4 °C. After three washes with TBST, the membrane was incubated with HRP (Cell Signaling) conjugated secondary antibody for 1 h. The FXII was detected with ELC substrate and image was captured with FluorChem E System (ProteinSimple). In some reactions, the activation of 100 nM FXII by 25 μg/ml LPS was evaluated similarly as described but using buffer supplemented with different NaCl concentration (10, 50, 100, or 150 mM). 5 μg/ml kaolin (Sigma) is very commonly used to experimentally trigger coagulation, was used as a positive control, while buffer only in the microtiter plate well was used as a vehicle control.

#### Continuous and discontinuous assays of FXII activation in a purified system

Continuous and discontinuous assays of 100 nM FXII activation by 25 μg/ml of O111:B4, O26:B6, or Rd2 aggregates were performed simultaneously in 20 mM Hepes buffer supplemented with 150 mM NaCl at 37 °C. In the discontinuous assay, aliquots were removed at specific times and transferred into buffer containing 0.1 mg/ml polybrene (hexadimethrine *bromide*; Sigma-Aldrich) to interrupt LPS−FXII interactions and quench the reactions. Subsequently, 300 μM S-2302 was added, and the amidolytic activities were measured. An identical procedure was used for the continuous assay, except polybrene was excluded from the reactions. FXIIa concentration at each quenched time point in the discontinuous assay was computed by measuring initial velocities of S-2302 hydrolysis and comparing these values to a standard curve generated with pure FXIIa. 5 μg/ml kaolin was used as a positive control, while buffer only was used as a vehicle control.

#### FXI autoactivation

To assess FXI autoactivation, 50 nM FXI (Prolytix) and 120 nM HK (Enzyme Research) and 0 or 2.5 μM CTI (Innovative Research) was dissolved in 20 mM Hepes buffer, pH 7.4, supplemented with 150 mM NaCl and preincubated with 25 μg/ml of O111:B4, O26:B6, or Rd2 aggregates for 2 h at 37 °C. Three hundred micromolars of the chromogenic substrate S-2366 (Chromogenix) was added and the amidolytic activity were then recorded using a plate reader. FXIa generation was monitored for 60 min at 37 °C. Five nanomolars FXIIa was used as a positive control, while buffer only in the microtiter plate well was used as a vehicle control.

#### PK autoactivation

To assess apparent PK autoactivation, 100 nM PK (Enzyme Research), 120 nM HK (Enzyme Research), and 0 to 3.5 μM CTI (Innovative Research) were dissolved in 20 mM Hepes buffer, pH 7.4, supplemented with 150 mM NaCl, and preincubated with 25 μg/ml of O111:B4, O26:B6, or Rd2 aggregates for 2 h at 37 °C. Three hundred micromolars of the chromogenic substrate S-2302 (Chromogenix) was added and PKa amidolytic activity were then recorded using a plate reader. PKa generation was monitored for 60 min at 37 °C.

#### Enzyme inhibition assays of purified proteins

100 nM FXIIa (Enzyme Research), 100 nM FXIa (Prolytix), or 100 nM PKa (Enzyme Research) and LPS aggregates (3–120 μg/ml) were placed into a 96-well plate in 20 mM Hepes buffer, supplemented with 150 mM NaCl at 37 °C. Amidolytic activities were recorded using the chromogenic substrate S-2302 (300 μM) for FXIIa/PKa and S-2366 (300 μM) for FXIa. Control samples with LPS and the substrate were used for background subtraction. Percent activities were calculated by dividing initial velocities (v_0_) at each LPS concentration by the v_0_ without the aggregates.

#### FXI and PK activation by LPS–FXII complex

50 nM PK (Enzyme Research) or 30 nM FXI (Prolytix) was placed into a 96-well plate in 20 mM Hepes buffer, supplemented with 150 mM NaCl at 37 °C with or without 50 nM FXII (Prolytix) and with or without 25 μg/ml of O111:B4, O26:B6, or Rd2 aggregates, and the amidolytic activity was quantified with 300 μM of the chromogenic substrate S-2302 for PKa or S-2366 for FXIa. Additional samples were removed at various times (0, 30, and 60 min), and the immunoblotting were analyzed with an HRP-conjugated polyclonal antibody against PK (Abcam) or FXI (Abcam) and image was captured with FluorChem E System (ProteinSimple).

#### Effects of “masked” monomeric LPS on FXII activation and FXIIa/FXIa/PKa regulation

For the preparation of “masked” monomeric LPS, 1 mg/ml of LPS chemotypes were spiked into 10 mM sodium citrate (Sigma), pH 7.5, 0.05% Triton X-100 (Sigma) and stored for at least 7 days at 4 °C (59). To assess the direct activation of FXII by LPS, 100 nM of FXII was preincubated with 25 μg/ml O111:B4, O26:B6, or Rd2 aggregates or “masked” monomers in 20 mM Hepes buffer, pH 7.4, supplemented with 150 mM NaCl, and the amidolytic activities were then recorded using a plate reader using 300 μM of the chromogenic substrate S-2302 for FXIIa/PKa or S-2366 for FXIa. The activity was monitored for 200 min at 37 °C. To assess the enzymatic regulation of the enzymes by LPS, 50 nM FXIIa or 50 nM PKa or 50 nM FXIa was incubated with 25 μg/ml O111:B4, O26:B6, or Rd2 aggregates or “masked” monomers in 20 mM Hepes buffer, pH 7.4, supplemented with 150 mM NaCl for 2 h at 37 °C. Amidolytic activities were then recorded using a plate reader using 300 μM of the chromogenic substrate S-2302 (Chromogenix). We observed no detectable enzymatic differences of FXIIa, FXIa, or PKa activities in control experiments supplemented with sodium citrate alone (data not shown).

### Effects of LPS chemotypes in human plasma

#### LPS characterization in human plasma

The dynamics of the LPS aggregation in plasma (Innovative Research) was monitored by DLS. Citrated human plasma was diluted 3-fold with Hepes buffer and incubated with 25 μg/ml LPS. DLS were recorded following an incubation time of 30 min. Results were compared against the DLS of LPS chemotypes diluted in pure buffer (control).

#### FXII/PK amidolytic activities in plasma

Citrated human plasma was diluted 3-fold in 20 mM Hepes buffer, pH 7.4, supplemented with 150 mM NaCl, and incubated with 25, 50, or 100 μg/ml of O111:B4, O26:B6, or Rd2 aggregates for 30 min at 37 °C. Hepes buffer without LPS was used as a vehicle control and 5 μg/ml Kaolin as a positive control. FXIIa/PKa amidolytic activities were recorded in a plate reader with the chromogenic substrate S-2302 (300 μM).

#### Investigation of LPS-induced coagulation in human plasma

Citrated human plasma was diluted 3-fold in 20 mM Hepes buffer, pH 7.4, supplemented with 150 mM NaCl, and incubated with 25, 50, or 100 μg/ml of O111:B4, O26:B6, or Rd2 aggregates for 5 min at 37 °C. After recalcification (6 mM CaCl2), time courses were recorded by turbidity measurements at 405 nm in a plate reader. Time to fibrin clot formation was determined from the intersection of the steep portion of the time course with the baseline absorbance.

## Data analysis

All data were analyzed with GraphPad Prism 9.4.1 (GraphPad Software, http://www.graphpad.com). Experiments were performed in triplicate, and results are presented as the mean SD of at least six separate repeats. Nonparametric one-way ANOVA, followed by the Kruskal–Wallis test, was used to compare group means; ∗*p* < 0.05, ∗∗*p* < 0.001 and ∗∗∗*p* < 0.0001 was considered as statistically significant.

## Data availability

All the data described in this work are contained within the manuscript and supporting materials.

## Supporting information

The supporting information is available free of charge at (to be added upon acceptance). This article contains supporting information.

## Conflicts of interest

C. U. L. and E. I. T. are employees of Aronora, Inc, a company that may have a commercial interest in the results of this research. J. J. S. serves as a medical consultant for Aronora, Inc. This potential conflict of interest has been reviewed and managed by conflict of interest in research committee of the Oregon Health & Science University. The remaining authors declare that they have no conflicts of interest with the contents of this article.

## Supporting information

The physicochemical properties of LPS
