# Supplementary material for: The physicochemical properties of lipopolysaccharide chemotypes regulate activation of the contact pathway of blood coagulation
Source: J Biol Chem. 2024 Dec 18;301(1):108110. doi: 10.1016/j.jbc.2024.108110 (PMC11773025; doi:10.1016/j.jbc.2024.108110)
Supplement: The physicochemical properties of LPS [file mmc1.pdf]

Supporting information

The physicochemical properties of lipopolysaccharide chemotypes regulate activation of the contact pathway of blood coagulation

André L. Lira[1], Berk Taskin[1], Cristina Puy[1], Ravi S. Keshari[2], Robert Silasi[2], Jiaqing Pang[1], Joseph E. Aslan[1,3], Joseph J. Shatzel[1,4], Christina U. Lorentz[1,5], Erik I. Tucker[1,5], Alvin H. Schmaier[6], David Gailani[7], Florea Lupu[2], Owen J. T. McCarty[1,3]*

List of included material:

Figure S1. Size distribution and hydrophobicity index of LPS chemotypes aggregates

Figure S2. Zymogens contact factors

Figure S3. The hydrodynamic diameter of FXII, FXI, PK and HK molecule

Figure S4. DLS analysis of LPS in the presence or absence of contact proteins

Figure S5. Fluorescence spectra for the direct interaction between FXII, FXI or PK and LPS

Figure S6. NaCl titrations of FXII activation by LPS aggregates

Figure S7. Size distribution and zeta potential of LPS in human plasma

Figure S8. Time course of fibrin formation in human plasma

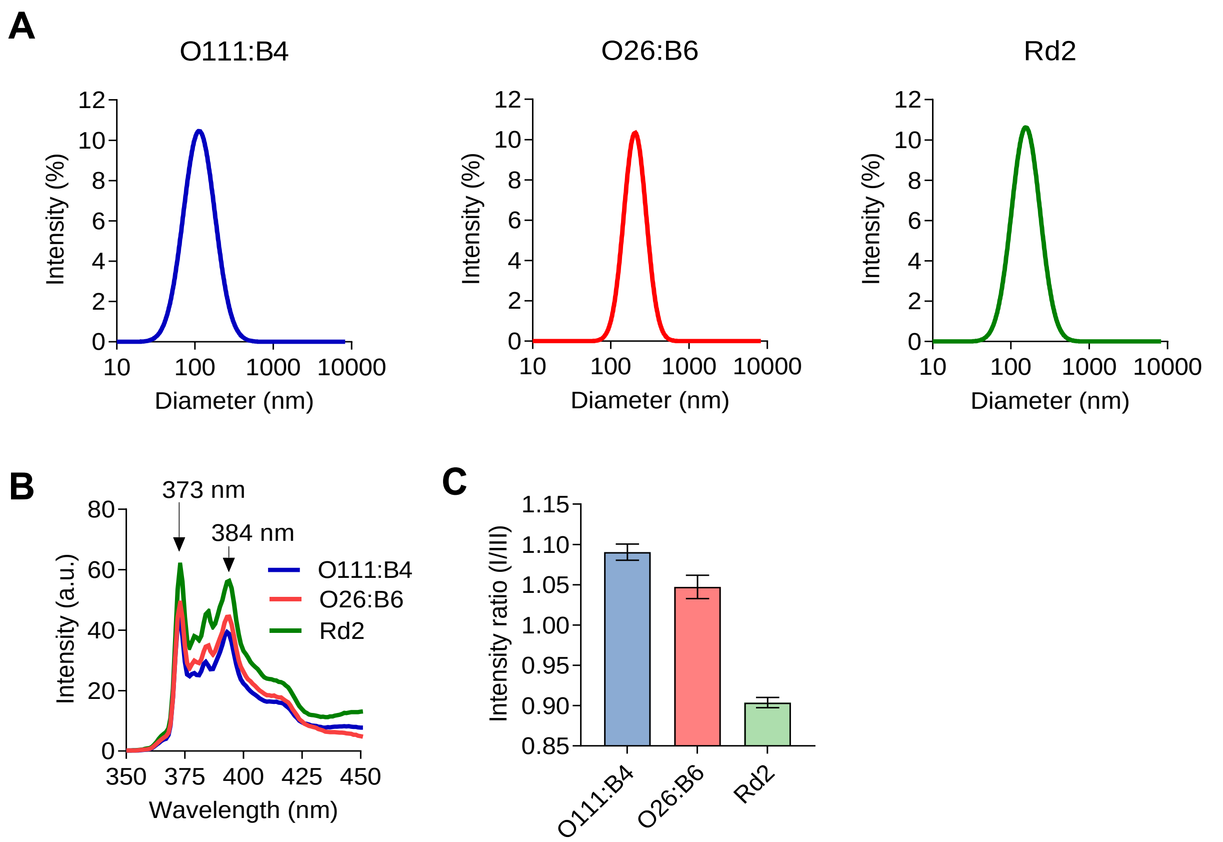

Figure S1. Size distribution and hydrophobicity index of LPS chemotypes aggregates. (A) The size distribution of LPS aggregates is shown for O111:B4 (blue), O26:B6 (red), and Rd2 (green), which peak with a d_h of 95 ± 6 nm, 196 ± 7, and 183 ± 11, respectively. The aggregates were analyzed in 20 mM HEPES buffer, pH 7.4. (B) The LPS hydrophobicity was analyzed by fluorescence employing pyrene as the probe molecule. Fluorescence emission spectra of pyrene (2.5 μg/mL) with LPS chemotypes O111:B4, O26:B6 or Rd2 (100 μg/mL) measured at a fixed excitation wavelength (λ_{ex} = 335 nm). Experiments were performed in HEPES buffer solution. (C) The hydrophobicity of LPS aggregates were characterized using pyrene as a fluorescent probe. Pyrene's emission spectrum features five peaks, with peak *I* increasing in intensity in polar solvents, while peak *III* remains unaffected. Changes in the intensity ratio of *I/III* provide insights into the polarity of the pyrene probe's surroundings, indicating the probe's containment within the LPS aggregate. Aggregate hydrophobicity index of the rough chemotype Rd2 was lower than for O111:B4 and O26:B6 (1.293 ± 0.011, 1.407 ± 0.026, and 1.453 ± 0.058; respectively). Bars are mean ± standard deviation (SD) of triplicate measurements.

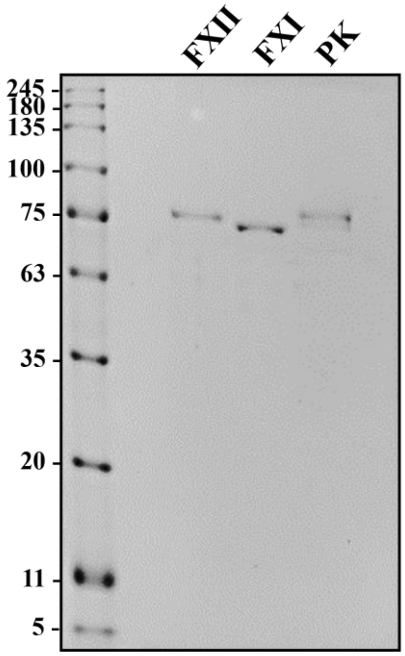

Figure S2. Zymogens contact factors. A Coomassie blue–stained non-reducing SDS-PAGE gel was utilized to analyze the proteins FXII, FXI, and PK, each loaded with approximately 1 μg per lane. Molecular mass standards, indicated in kilodaltons, are positioned to the left of the gel images for reference.

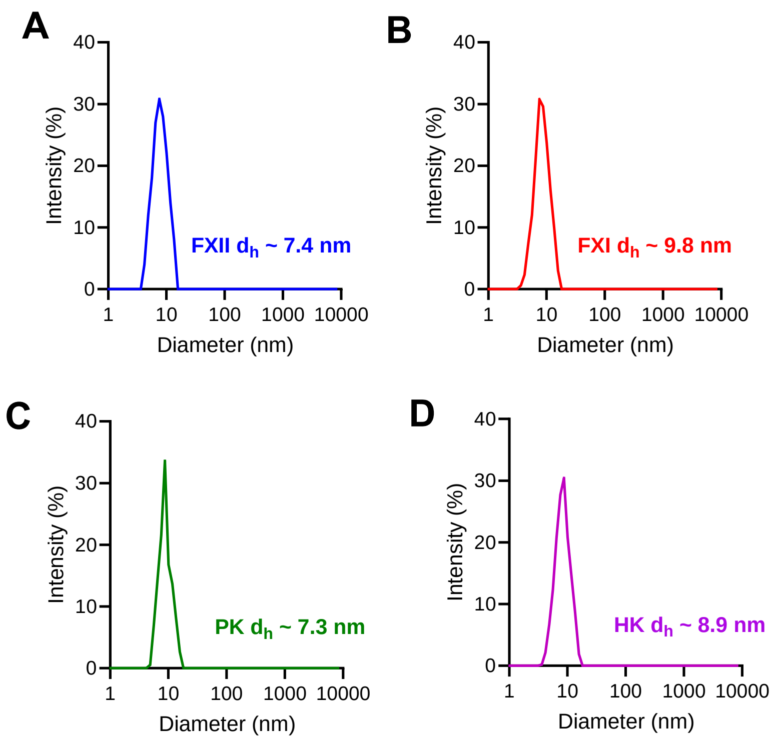

Figure S3. The hydrodynamic diameter of FXII, FXI, PK and HK molecule. Dynamic light scattering (DLS) was conducted at 25°C with a protein concentration of 10 nM.

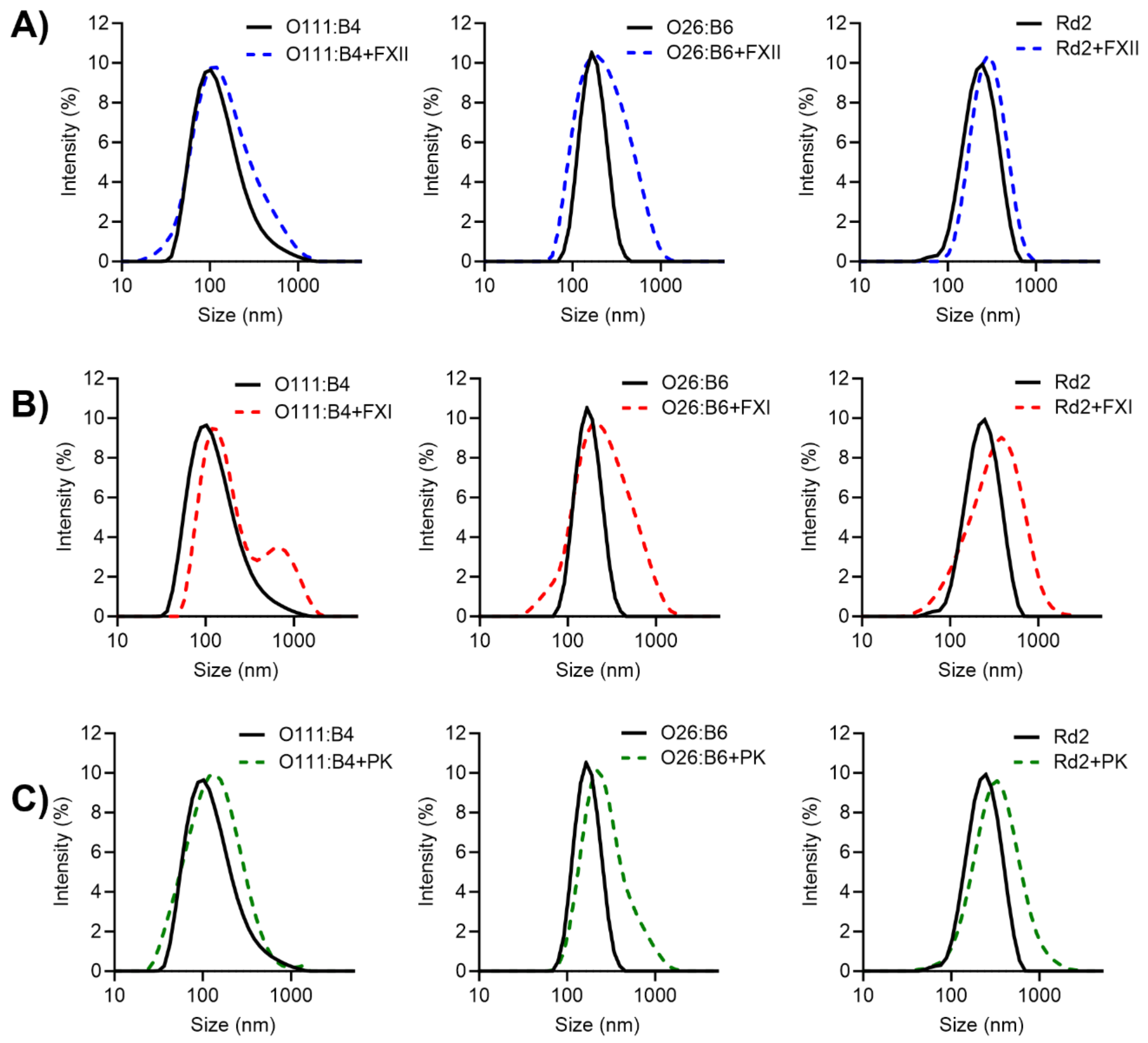

Figure S4. DLS analysis of LPS in the presence or absence of contact proteins. The size distribution of LPS in buffer is shown as a solid line which peaked with a size of 95, 196 and 183 nm for O111:B4, O26:B6 and Rd2, respectively. (A) After incubation with FXII the size increased to 127, 231 and 215 nm for O111:B4, O26:B6 and Rd2, respectively (blue dashed line). (B) After incubation with FXI the size increased to 143, 247 and 264 nm for O111:B4, O26:B6 and Rd2, respectively (red dashed line). (C) After incubation with PK the size increased to 150, 242 and 226 nm for O111:B4, O26:B6 and Rd2, respectively (green dashed line).

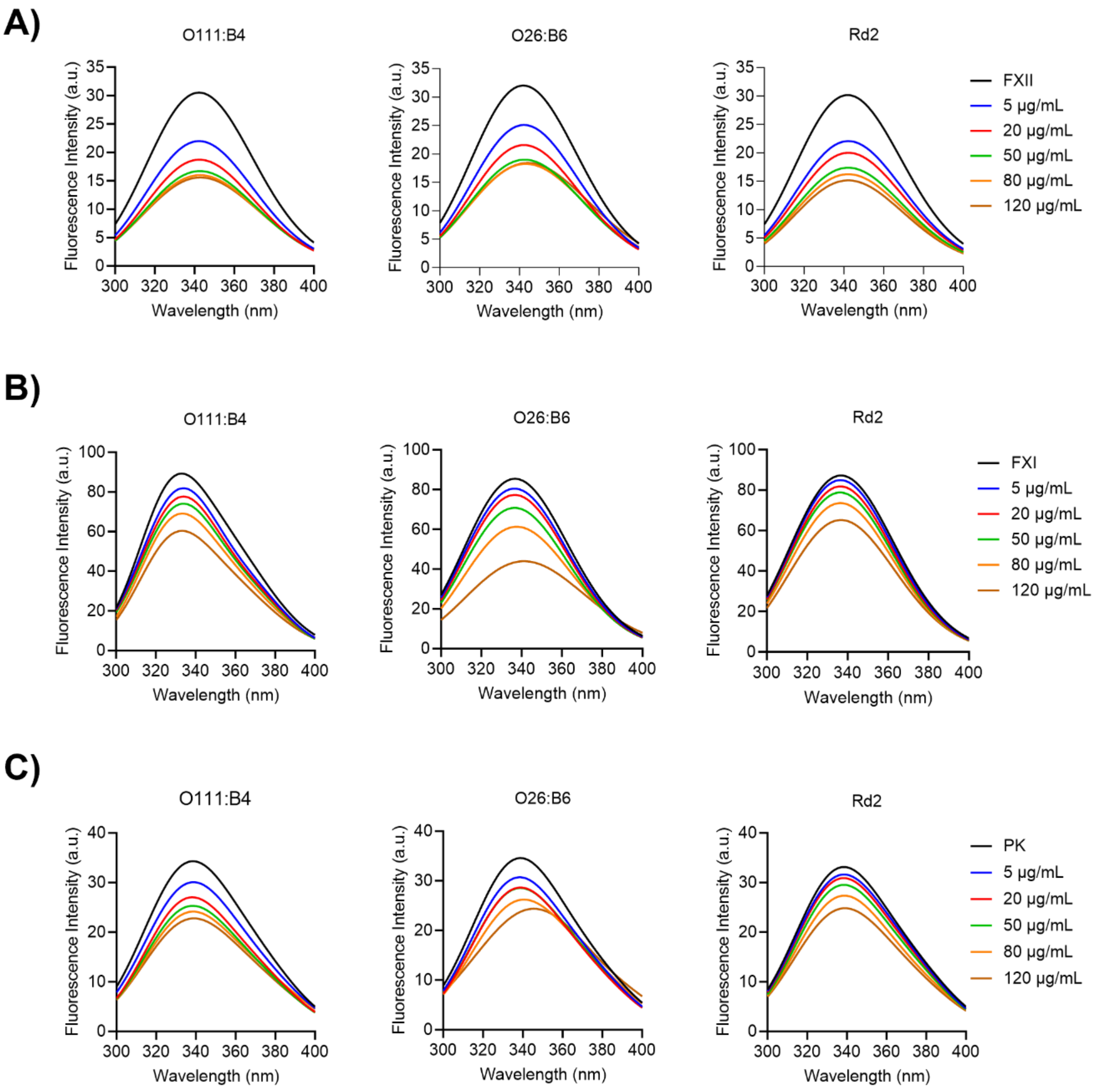

Figure S5. Fluorescence spectra for the direct interaction between FXII, FXI or PK and LPS. The tryptophan fluorescence spectra of FXII, PK or FXI (0.5 µM), excited at 280 nm, exhibited a reduction in intensity upon titration with various concentrations of LPS chemotypes. This decrease in fluorescence reached saturation when LPS concentrations were increased to approximately 50 µg/mL. Data are expressed as mean ± SD of three independent experiments.

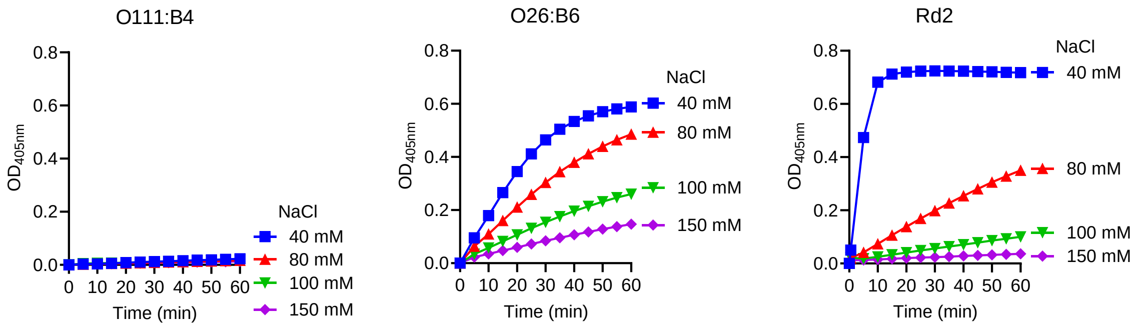

Figure S6. NaCl titrations of FXII activation by LPS aggregates. FXII (100 nM) was incubated with LPS aggregates (50 μg/mL) in HEPES buffer containing 40–150 mM NaCl. FXII activation was followed in time using the chromogenic substrate S2302. Initial rates of FXII activation were plotted as a function of NaCl concentration. The concentration of NaCl in the reaction had a dramatic impact on autoactivation of FXII by LPS. It was found maximal autoactivation of FXII by O26:B6 and Rd2 at lowers NaCl concentrations; however, considerable activity in the presence of O26:B6 was still detectable at physiological NaCl concentrations. Nevertheless, it was not detected amidolytic activity in the presence of O111:B4 in any concentration of NaCl. The data points represent the averages of triplicate measurements, with error bars indicating ±1 standard deviation (SD).

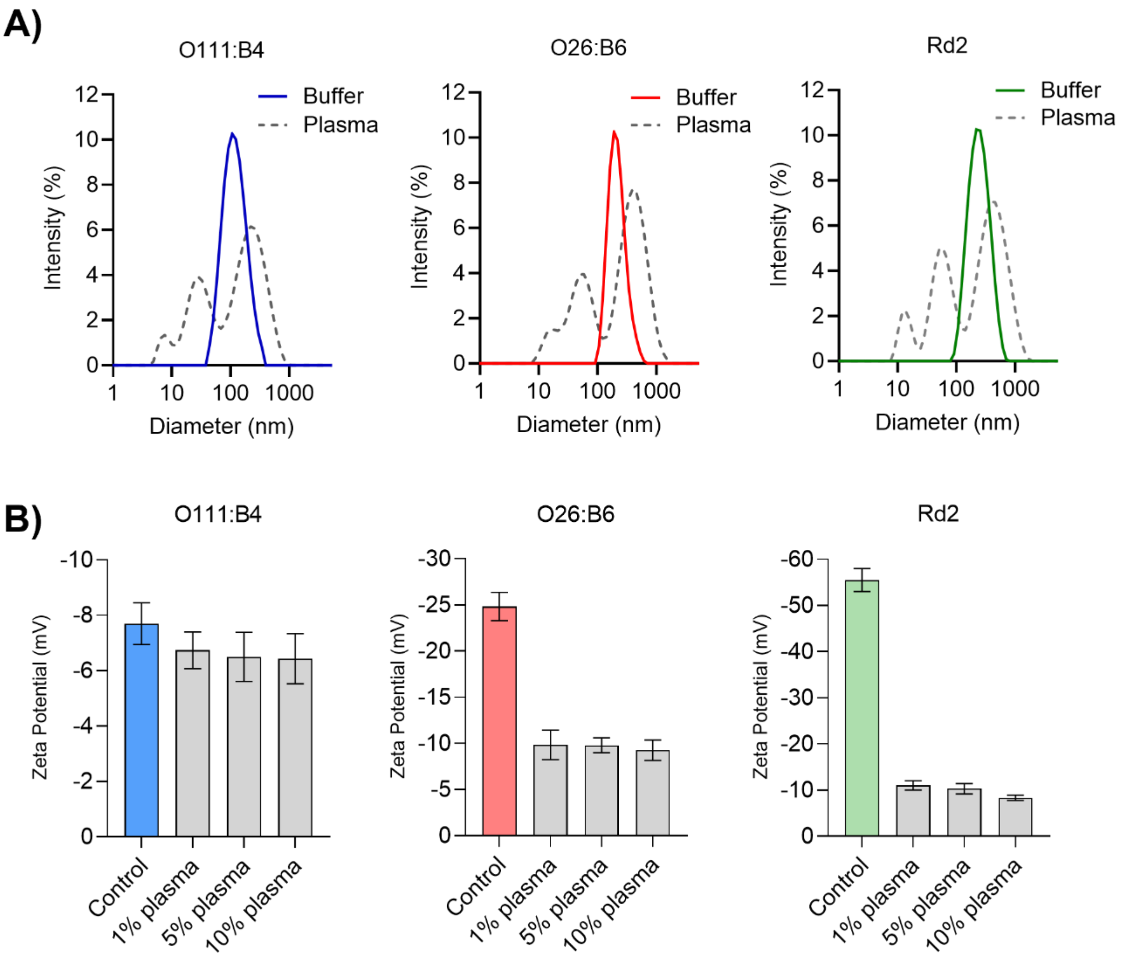

Figure S7. Size distribution and zeta potential of LPS in human plasma. (A) LPS size distribution plot of aggregates in buffer and human plasma. The displayed size disparity between aggregates incubated in buffer and plasma might occur due the disaggregation effects caused by some proteins such as albumin, alongside the formation of a biomolecular corona. (B) ZP of LPS chemotypes after exposure to different amounts of human plasma. The data points represent the averages of triplicate measurements, with error bars indicating ±1 standard deviation (SD).

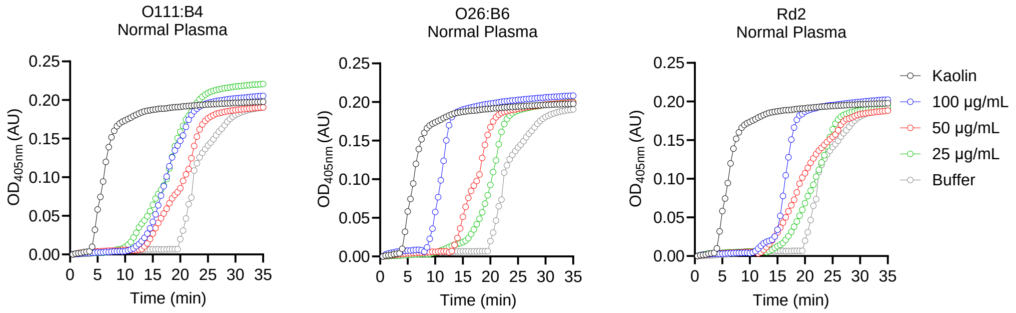

Figure S8. Time course of fibrin formation in human plasma. Assessment of procoagulant activity of LPS chemotypes. Citrated normal plasma was diluted 3-fold and mixed with or without O111:B4, O26:B6 or Rd2 (25, 50, 100 μg/mL) for 5 min at 37°C. After recalcification, time courses were recorded by turbidity measurements at 405 nm.
